# Effect of Approach Distance and Change of Direction Angles Upon Step and Joint Kinematics, Peak Muscle Activation, and Change of Direction Performance

**DOI:** 10.3389/fspor.2020.594567

**Published:** 2020-10-30

**Authors:** Hallvard Nygaard Falch, Håvard Guldteig Rædergård, Roland van den Tillaar

**Affiliations:** Department of Sport Sciences and Physical Education, Nord University, Levanger, Norway

**Keywords:** change of direction, strength-dominant, velocity-dominant, EMG, COD performance

## Abstract

The aim of the study was to compare the step kinematics, joint angles, and muscle activations between change of direction (COD) maneuvers with different angles and approach distances, suggested to require different strength and velocity demands. Twenty-three male soccer players completed eight COD tests consisting of both 4 and 20-m sprint approaches with one directional change which varied between each COD test (45, 90, 135, and 180°). Peak muscle activity, step and joint kinematics of the lower limbs of the plant, and re-acceleration step were measured. Compared to 4-m CODs, the 20-m COD approach distances increased vertical center of mass displacement (*p* < 0.001), number of deceleration steps (*p* < 0.001), revealing no statistical differences upon joint angles (*p* > 0.05). Greater COD angles resulted in increased ankle dorsiflexion, hip abduction and flexion, greater displacement of the center of mass and tibia angle, longer contact times, and more deceleration steps (*p* < 0.034). The CODs categorized as velocity-dominant revealed higher peak muscle activity in the adductor longus, semitendinosus, biceps femoris, and gastrocnemius. It was concluded that velocity-dominant CODs revealed higher muscle activity due to a higher eccentric loading, implicating task-specific training considerations for enhancing COD performance.

## Introduction

Field sports require many different abilities for success (Reilly et al., 2000; Stølen et al., 2005). One of those abilities is to move fast, because fast movements are often decisive for the match-outcome in sports such as soccer (Helgerud et al., 2001; Faude et al., 2012). The ability to change direction fast in field sports are characterized by the athletes' change of direction- (COD) ability which is important for creating space in offensive play. Brughelli et al. (2008) defined COD as a pre-planned whole-body movement into a new direction, thus being greatly dictated by the athlete's physical abilities, rather than perceptual and decision-making factors (Young et al., 2002).

CODs consists of an acceleration phase, which is similar to the acceleration phase in a sprint, where the athlete's center of mass is first lowered to produce horizontal force to the ground. This is typically followed by a deceleration/braking phase caused by eccentric muscle work (Jones et al., 2017) and a directional change (turn) that involves a manipulation of the base of support relative to the center of mass to apply an external force to reaccelerate into a new direction (Spiteri et al., 2013; Jones et al., 2017). Generally, the turn involves a lateral/anterior foot plant (herein referred to as the plant step) that requires both a braking and propulsive force components (Dos'Santos et al., 2018). Subsequently, a new acceleration in the new direction is performed.

Analysis of soccer matches showed that soccer players perform about 700 turns per game at different intensities, turning either right or left (Bloomfield et al., 2007). Reilly et al. (2000) found COD-performance to be a discriminating variable when comparing young elite and sub-elite soccer players. Thus, how to improve COD performance is of great interest for strength and conditioning coaches. COD is a task-specific skill (Young et al., 2001; Sheppard and Young, 2006), with varying requirements across distinctive sports due to different sprint lengths and angle of the CODs occurring. In a review, Dos'Santos et al. (2018) highlighted that angle of the turn and velocity approaching the COD are determinants affecting decelerating and re-acceleration requirements when performing a COD (angle-velocity trade-off). Faster approaching velocities limits the subsequent COD due to greater braking required to change the momentum from higher velocities, while the angle of the COD sets limitations of the approaching velocity (Dos'Santos et al., 2018). The predictors for COD performance are shown to vary by angle of the COD as the angle affects the technique for executing the task. Bourgeois et al. (2017) suggest that the angle of the COD and velocity approaching the plant step demands different strength and velocity requirements of the lower limb muscles when rapidly redirecting the body to change momentum in a COD. Sharper CODs (>90°) are suggested to be more dependent on strength (force dominant), opposed to velocity CODs (<90°) which are suggested to be more velocity based.

The sharper cuts require the plant foots' center of pressure to be more laterally orientated relative to the center of mass to generate greater mediolateral forces (Havens and Sigward, 2015). Furthermore, sharper angles demand greater redirections of the whole body (Havens and Sigward, 2015), inducing reduced velocities and increasing stance times in both the penultimate step and in the plant step, suggesting greater braking and translation to manage the turn in sharper CODs. Furthermore, Schot et al. (1995) observed greater average braking forces in the sharper COD (45 vs. 90°) as the 45° COD allows for greater maintenance of velocity by transferring motion into the new direction, rather than absorbing it (Dos'Santos et al., 2018).

Similar explanations have also been applied for explaining the different muscle activity observed in earlier research. When comparing EMG responses for the vastus lateralis and biceps femoris in a 45 vs. 90° COD, Hader et al. (2016) found the 90° COD to elicit greater muscle activity. Thereby, suggesting muscle activity to be angle dependent, with activity increasing with increasing angle, due to greater requirements of braking and re-accelerating. Likewise, Besier et al. (2003) found greater muscle activity in the plant step of 30 and 60° angle CODs compared to straight-line running when averaging the muscle activity from 10 lower limb muscles surrounding the knee. However, in all these studies only CODs with change of direction angles of 90° and less were compared with each other.

**Figure 1:**
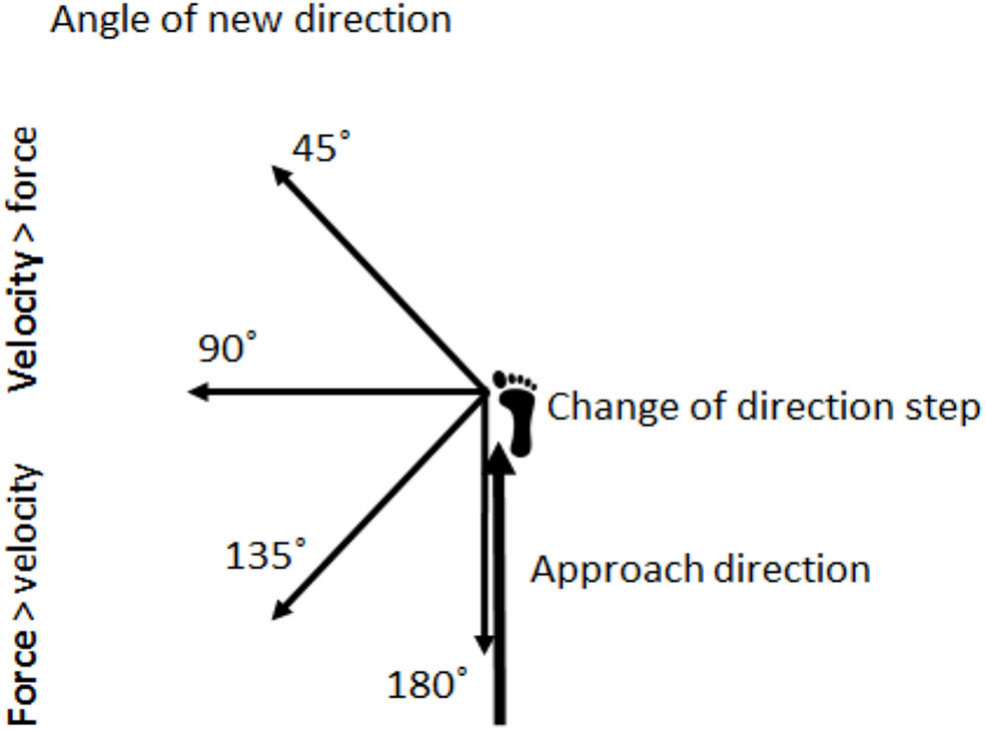
Illustration of how the entering speed in the approach direction and angles of the plant step make the COD velocity-dominant or force-dominant. The figure is adapted from Bourgeois et al. (2017).

Furthermore, research investigating how differences in strength and velocity requirements in terms of angles and approaching velocities affects step and joint kinematics and muscle activation is inadequate, as the literature is greatly stemmed from simple comparisons (two different approaching distances or two different COD angles). Only Schreurs et al. (2017) investigated CODs with 45, 90, 135, and 180° angles, but from only one approach distance. In their study, both kinematics and kinetics was collected, but no muscle activation was collected. Therefore, the objective of the current study was to compare joint angles and step kinematics in CODs from different approaches in angles suggested to require different strength and velocity demands. An additional objective was to compare muscle activation in the suggested force vs. velocity-dominant CODs (Figure 1). The comparison could implicate training considerations by providing valuable information about the technical requirements of the distinctive CODs, which is crucial when seeking to develop sport-specific COD performance.

Higher muscle activity in the lower limb muscles in force-dominant CODs than in velocity-dominant CODs was expected, as increased angle of directional change been found to increase muscle activity in earlier studies (Besier et al., 2003; Hader et al., 2016). Furthermore, contact time, center of mass displacement, and number of deceleration steps were hypothesized to increase due to greater braking requirements. With respect to approach distance, higher muscle activation was hypothesized in smaller angles of direction change (<45°) as players are expected to continue increasing velocity throughout the task. 20-m CODs were also expected to induce a higher number of braking steps compared to their 4-m counterpart due to increased braking requirements a priori the plant step.

## Method

To compare step and joint kinematics and muscle activation in the different CODs, a within-subject design was used to determine the effect of angles and approach distance upon the variables related to COD performance. Twenty-three male soccer players volunteered to participate in this study.

### Participants

Twenty-three experienced Norwegian male soccer players (age: 22.5 ± 2.6 years, height: 181.4 ± 6.3 cm, body mass: 79.6 ± 8 kg, 2nd−6th national playing level) participated in this study. The requirement for participation was a minimum of two soccer-training sessions a week. All participants preferred the right foot as the kicking foot, hereby referred to as the dominant foot. Previous research has shown that performing the plant step with preferred kicking foot is superior in terms of COD performance (Rouissi et al., 2016). Each participant was informed of the testing procedures and possible risks, and written consent was obtained prior to the study. The study complied with the current ethical regulations for research and was approved by the regional ethics committee, conforming to the latest revision of the Declaration of Helsinki. The participants were informed not to consume alcohol 24 h prior to testing and to eat a light meal 2 h before the start of the test.

### Procedures

Participants took part in two familiarization sessions in which the first took place 2–3 weeks before the test day. In the familiarization sessions, they practiced the COD test to minimize a possible learning effect. In the first familiarization session, the participants came in groups before answering a questionnaire consisting of questions about training sessions per week, dominant foot, and the level of playing. During the familiarization sessions, the performances in a COD test were noted and used to motivate and control for maximum effort during the test day. Participants were encouraged to improve performance from each familiarization session and on the day of testing. The warm-up protocol was the same for familiarization sessions and test days, but the order of the different CODs was always randomized to prevent the results from being affected by the test order. On the test day, the participants were tested one by one. The test day started with the subject's height being taken, followed by being weighed on a standing scale (Soehnle professional 7830, stand scale). Afterwards, 10 electromyography (EMG) electrodes were placed on the participant's dominant foot side, and the participant put on a full body motion capture suit.

Subsequently, the warm-up for the COD test started which consisted of 5 min of jogging, followed up by three runs at different intensities (60, 70, and 80% of self-perceived max speed). Then four sprints of 15 m, with a 65 and 110° right and left turn, were performed at self-assumed 80% of maximal effort. The specific warm-up was included for the participants to repeat the test, without favoring any CODs, before performing the test at maximum effort. Between each run during the warm-up, 60-s rest was included to minimize fatigue. The COD tests was performed with a 3–5-min rest between each run to avoid fatigue.

**Figure 2:**
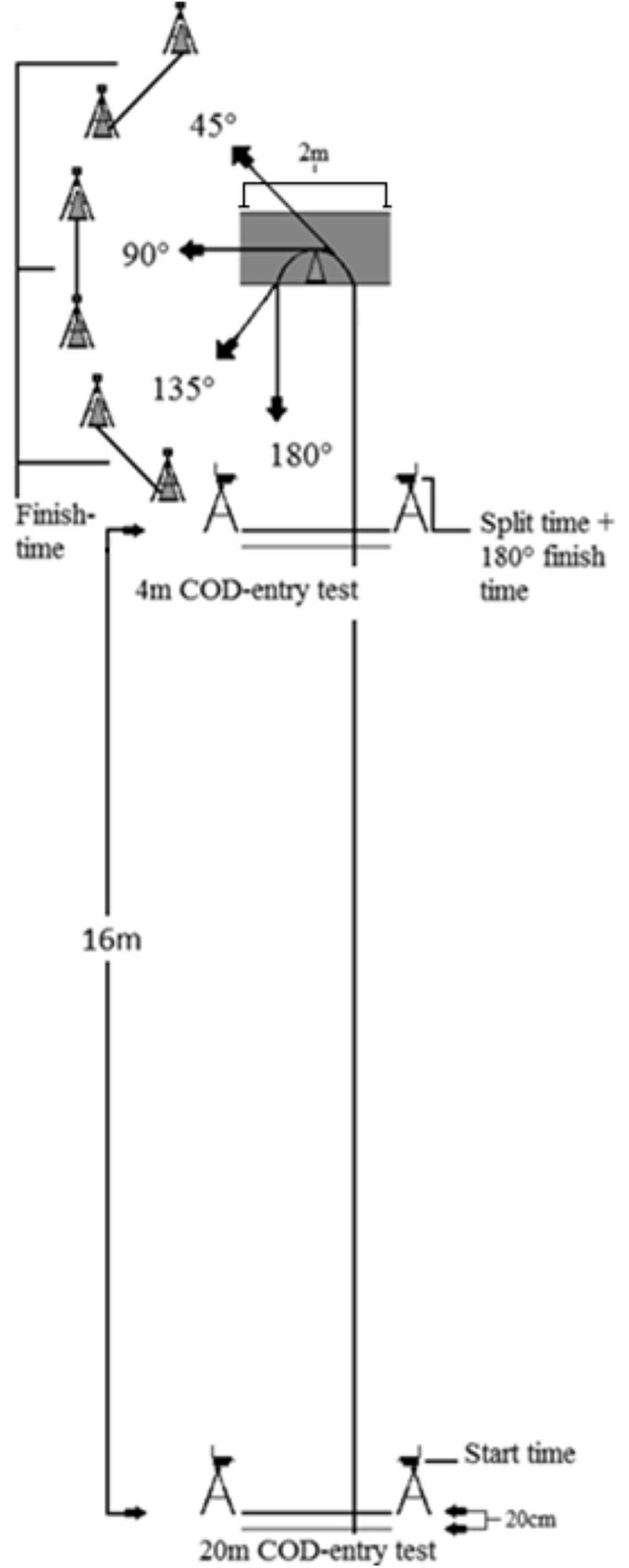
COD tracks. Illustration of dimensions for setup and testing.

To measure the technical and muscular variables related to COD performance without the aerobic systems functioning as a limiting factor for performance outcomes, the COD test was short, with only one turn (Brughelli et al., 2008). Therefore, the COD test consisted of a length of both 4 and 20-m to separate the extremities of velocities in the sprint when approaching the COD, followed by a 4-m re-acceleration toward a new direction to complete the test (Figure 2). The COD test was based on a similar approach of Schreurs et al. (2017).

The athletes were instructed to complete each COD run within the shortest total time possible. The COD test was performed on an indoor court surface (Taraflex Sport Evolution M 7.0 mm, Unisport, Finland). All CODs were performed from either 20 or 4-m with a left turn, where the angle of direction change was 45, 90, 135, or 180°, making it eight CODs in total in which the dominant foot of the participants (right) was used as the plant step. Each COD started with a standing start with the front foot placed 20 cm behind the timing gates, which was placed on each side of a 2-m long line. The timing gates were set with a height of 30 cm to prevent the upper limbs from starting the timer before participants initiate the sprint. Timing gates for measuring partial time and finishing of the test were set at a height of 95 cm. This ensured that timing gates were set at approximately hip height and time measurement would not be determined by random extension of limbs. For an attempt to be approved, the subject had to perform the COD with both feet inside the COD area, without overstepping the rear end of the area or the turning cone (Figure 2), except for the 45° COD. The turning cone was removed when performing the 180° COD. The participants had one attempt at each condition, but in case of slipping or violations of the test requirements, one extra attempt was performed. Although it was rarely required, a test attempt resulting in a performance decrease of 0.1 s or more from the second familiarization day resulted in a re-attempt to ensure maximum performance.

### Measurements

The time to perform the COD test was measured for both total time and partial time using timing gates. A wireless timer (Brower timing systems, Salt Lake City, Utah, USA) registered total time and partial time. Total time was the time to complete the test (4 or 20-m + COD + 4-m), while partial time was the split time when performing the 20-m CODs (last 4-m before COD + COD + 4-m) (Figure 2). The participants were not informed about the measurement of partial time in order to prevent them from beginning the COD at a low velocity, making the COD more match-like. Both total and partial times were used for statistical analysis.

Muscle activity was measured by using a wireless EMG with a sampling rate of 1 kHz (Ergotest Innovation, Porsgrunn, Norway) with electrodes (Zynex Neurodiagnostics, CO, USA) on the muscles of the right leg. Before fastening the electrodes, the skin was shaved and cleaned with alcohol on the following muscles: lateral and medial vastii, rectus femoris, adductor longus, biceps femoris, semitendinosus, soleus, lateral gastrocnemius, gluteus medius, and maximus muscles. The electrodes (11-mm contact diameter and 2-cm center-to-center distance) were placed along the presumed direction of the underlying muscle fibers, according to the recommendations of Hermens et al. (2000). The EMG raw signal was amplified and filtered using a preamplifier located as close as possible to the pickup point with the intention of minimizing the noise induced from external sources through the signal cables. The preamplifier had a common mode rejection ratio of 100 dB. The EMG raw signal was then bandpass filtered (fourth-order Butterworth filter) with cut-off frequencies of 20 and 500 Hz, rectified and smoothed (moving average filter, 50 ms window width) (Hermens et al., 2000). The highest observed EMG signal for each muscle in the plant step and the first acceleration step performed by the dominant foot in each COD was used for further analysis.

**Figure 3:**
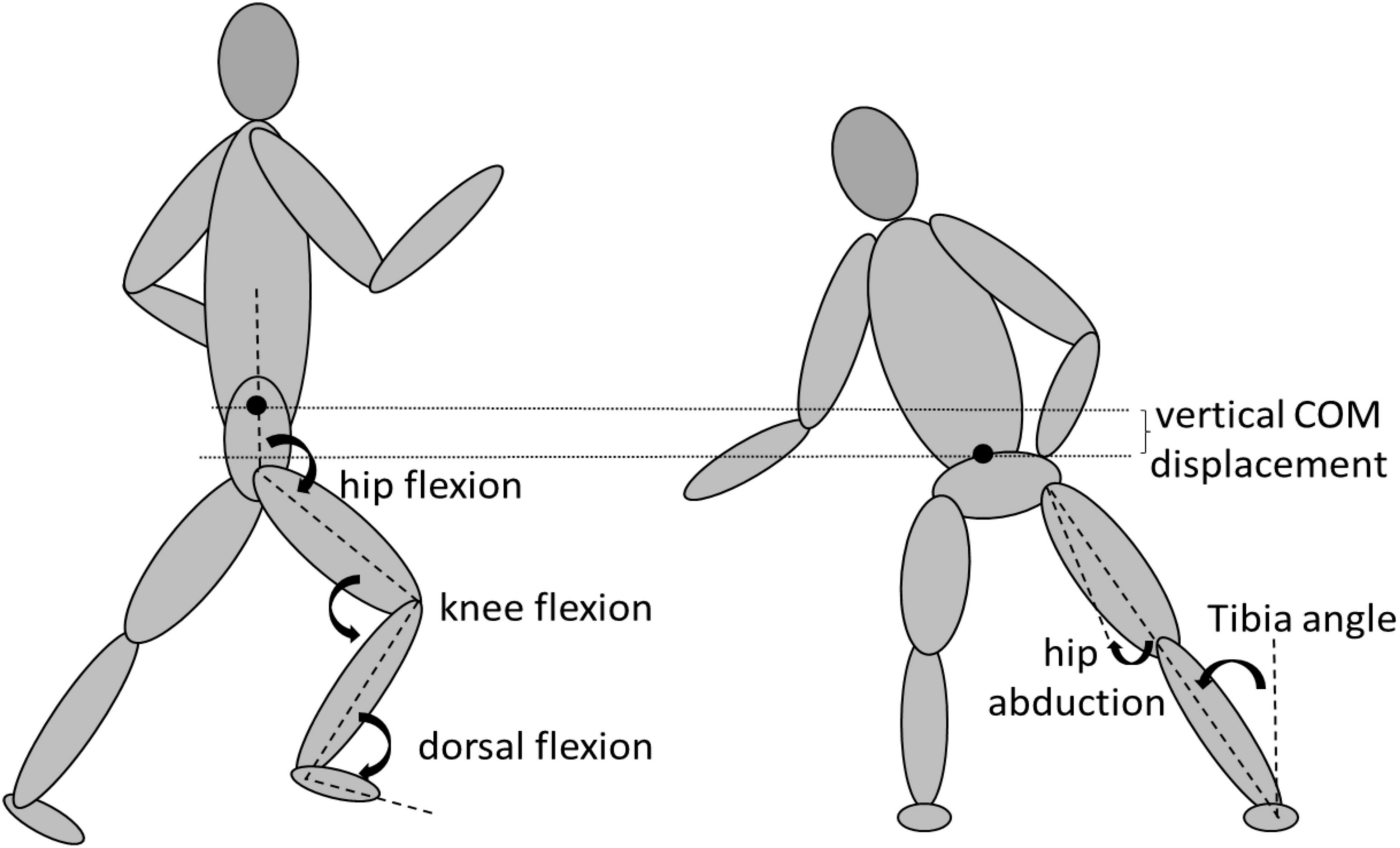
Definition of COM displacement, ankle dorsiflexion, knee flexion, hip flexion and abduction, and tibia angle angles during COD.

The EMG collected in Musclelab 10.5.69.4823 (Ergotest innovation A.S, Porsgrund, Norway) was synchronized with a 3D motion-capture system, Xsens motion capture, MVN link (Xsens Technologies B.V. Enschede, Netherlands). Xsens is a full-body motion-capture system, based on inertial sensors with a sample rate of 240 Hz, which creates a biomechanical model using 17 IMUs placed on different anatomical points derived from the user manual of Xsens. Modeling procedures for the inertial sensor data was based on the manufacturer's recommendations using the manufactures proprietary algorithms (Xsens Kalman Filter) and filtered using the LXsolver (minimize soft tissue artifact) in MVN Biomech Studio (version 2019.3). The system showed very high reliability with other optical 3D motion capture systems (Blair et al., 2018). By using the changes in velocity of the pelvis and the reduction in height of the center of mass, the number of braking steps when performing a COD was detectable in Xsens V.19.3. Deceleration steps were manually counted from the first step of decreasing pelvis' velocity, which simultaneously increased center of mass displacement, until the plant step (if a reduction in pelvis' velocity did occur in the plant step). Also, the lowest center of mass, defined as the largest displacement of the center of mass in the plant step with the dominant foot still in contact with the ground, was found using Xsens. In the plant step and first re-acceleration step while performing a COD, contact time was measured by the IMU on the plant foot by an algorithm (sudden decrease and increase in angular velocity of IMU on top of the plant foot) based upon pilot data and visually verified from the first frame in Xsens where the dominant foot was in contact with the ground, to the last frame where the dominant foot was still in contact with the ground. Joint angles (ankle dorsiflexion, knee flexion, hip flexion, and abduction) from the right limb were derived from the plant step, at the largest displacement of the center of mass because at this point most joint movements change from flexion to extension in which many muscles change their role from eccentric to concentric work. The tibia angle of the subject in the plant step was derived from the dominant lower leg and the line perpendicular to the floor (Figure 3). The number of deceleration steps, lowest center of mass, contact time in the plant, and acceleration steps and joint angles measured were used for further statistical analysis.

### Statistical Analysis

A 2 (approach distance: 4 vs. 20-m) x 4 (COD angles: 45, 90, 135, 180°) analysis of variance (ANOVA) with repeated measures was used. If significant differences were found, a one-way repeated measures ANOVA was also used to analyse the effect of angle on the performance-related variables center of mass, contact time, and joint angles when performing the different CODs (distance and angles). EMG measurements were categorized in force-dominant (135 and 180°) and velocity-dominant (45, 90°) CODs and a 2 (approach distance: 4 vs. 20-m) x 2 (force vs. velocity dominant) repeated measures ANOVA was performed to compare muscle activation between the force and velocity dominant CODs. When significant differences were found, Holm–Bonferroni *post-hoc* tests were conducted. When the assumption of sphericity was violated, the Greenhouse–Geisser correction was reported. The effect of the different variables is presented as partial eta squared (η^2^) where 0.01 < η^2^ < 0.06 constituted a small effect, 0.06 < η^2^ < 0.14 a medium effect, and η^2^ > 0.14 a large effect (Cohen, 1988). The alpha level was set at *p* < 0.05.

## Results

### Step Kinematics

A statistically significant effect of angle of the COD turn was observed for time to complete the COD test [*F*_(3,66)_ ≥ 719; *p* < 0.01; η^2^ = 0.97], displacement of the center of mass [*F*_(2.56,57.8)_ =100; *p* < 0.01; η^2^ ≥ 0.85], contact time in the acceleration and plant step [*F*_(2.88,58.3)_ ≥ 20.6; *p* < 0.01; η^2^ ≥ 0.58], and number of deceleration steps [*F*_(3,66)_ = 180; *p* < 0.01; η^2^ = 0.90]. Furthermore, a statistically significant effect of approach distance upon displacement of the center of mass [*F*_(1,22)_ = 11.45; *p* = 0.003; η^2^ = 0.39] and number of deceleration steps was found [*F*_(1,22)_ = 474; *p* < 0.01; η^2^ = 0.96]. *Post-hoc* comparison revealed an increase of time to complete the COD test when the angle of the plant step increased (Table 1). The number of deceleration steps increased for all CODs when compared to the 20-m condition and when the angle of the plant step increased (except for 135 vs 180°). The displacement of the center of mass in the plant step was significantly different between 45 and 90° COD from all other CODs (Table 1). The 4-m 45, 90, and 135° CODs also revealed significantly lower displacement of the center of mass when compared to the 20-m condition. The contact time in the plant step increased for both the 4 and 20-m CODs when the angle of the plant step increased. The contact time in the 4-m 45° acceleration step was shorter compared to the acceleration step in the 4-m 135 and 180° CODs, while the 20-m 45° acceleration step revealed shorter contact time compared to the acceleration step in all other CODs from the same distance. The acceleration step in the 20-m 90° COD was found to be shorter compared to the acceleration step when performing the 180° COD from the same distance. No statistically significant interaction effects (angle x distance) were observed for any of the measured variables [*F*_(1,22)_ ≤ 1.75; *p* > 0.17; η^2^ ≥ 0.08].

**Table 1:** Mean (± SD) descriptive statistics of the measured variables when performing a COD at different degrees with a 4 and 20 m approach.

	**45°**	**90°**	**135°**	**180°**
COD time 4 m (s)	1.70 ± 0.16^↓^^*^	2.04 ± 0.15^↓^^*^	2.36 ± 0.16^↓^^*^	2.48 ± 0.15^↓^^*^
Partial time 20 m (s)	1.38 ± 0.1^*^	1.83 ± 0.11^*^	2.15 ± 0.13	2.30 ± 0.12
COD time 20 m (s)	4.04 ± 0.19^*^	4.53 ± 0.19^*^	4.85 ± 0.23^*^	4.99 ± 0.26^*^
Deceleration steps 4 m (*n*)	0.4 ± 0.8^↓^^*^	2.7 ± 0.9^↓^^*^	3.3 ± 0.5^↓^	3.3 ± 0.7^↓^
Deceleration steps 20 m (*n*)	2.9 ± 1.1^*^	5.4 ± 0.8^*^	6.4 ± 1.3	6.3 ± 1
Center of mass plant step 4 m (m)	0.18 ± 0.03^↓^^*^	0.26 ± 0.05^↓^^*^	0.3 ± 0.06^↓^	0.33 ± 0.07
Center of mass plant step 20 m (m)	0.20 ± 0.03^*^	0.29 ± 0.04^*^	0.32 ± 0.06	0.35 ± 0.06
Contact time plant step 4 m (s)	0.15 ± 0.02^*^	0.18 ± 0.04^*^	0.23 ± 0.07^*^	0.30 ± 0.09^*^
Contact time plant step 20 m (s)	0.15 ± 0.02^*^	0.18 ± 0.04^*^	0.21 ± 0.0^*^7	0.33 ± 0.01^*^
Contact time acceleration-step 4 m (s)	0.16 ± 0.04^‡^	0.18 ± 0.06	0.20 ± 0.03	0.20 ± 0.04
Contact time acceleration-step 20 m (s)	0.15 ± 0.02^*^	0.18 ± 0.03^†^	0.20 ± 0.03	0.21 ± 0.04^†^

* *indicates a significant difference with all other angles on a p < 0.05 level*.

† *indicates a significant difference between these two angles on a p < 0.05 level*.

‡ *indicates a significant difference 45° angle with the 135° and 180° on a p < 0.05 level*.

↓ *indicates a significant difference between the 4 and 20m condition with this degree of the COD-turn*.

### Joint Kinematics

**Figure 4:**
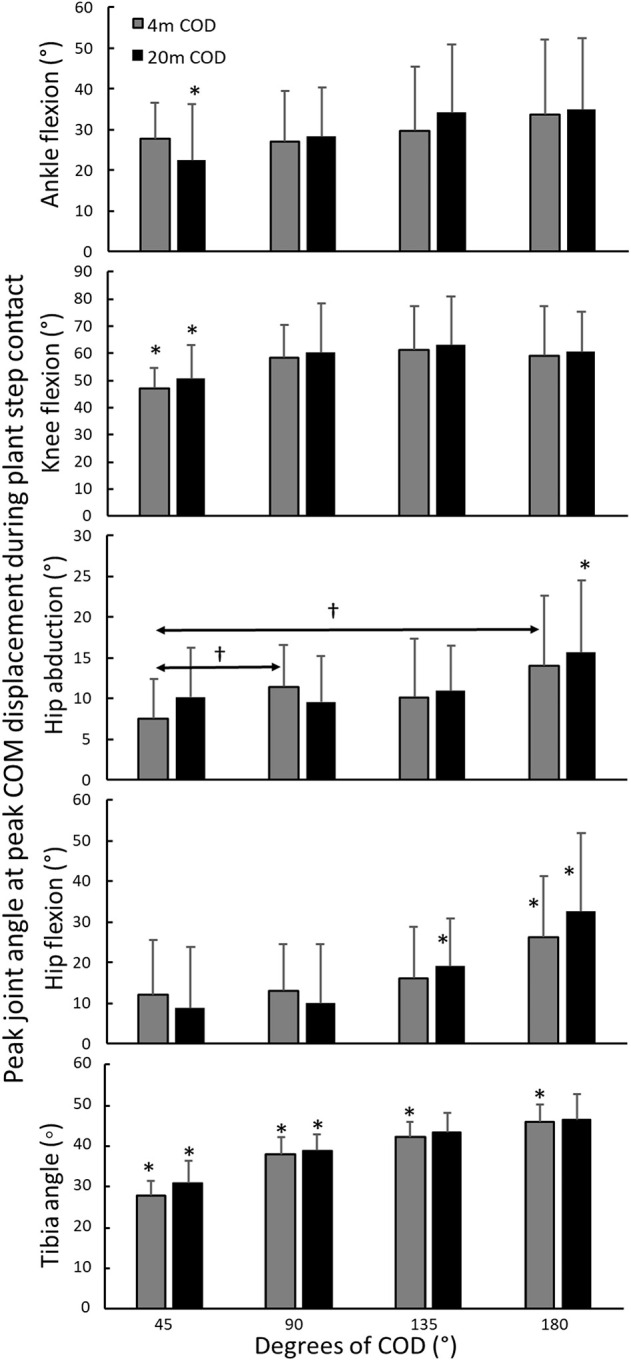
Comparison of joint angles (±SD) in the plant step when performing CODs with 4 and 20-m sprint approaches at different COD angles. ^*^Statistically significantly different compared to all other CODs from the same distance. ^†^Statistically significant difference between these two CODs for this approach condition.

A significant effect of angle was observed for tibia angle [*F*_(2.6,54.6)_ = 114; *p* < 0.01; η^2^ = 0.88], ankle dorsiflexion [*F*_(3,66)_ = 4.6; *p* = 0.012; η^2^ = 0.21], knee flexion [*F*_(3,66)_ = 9.0; *p* < 0.01; η^2^ = 0.32], hip abduction [*F*_(3,66)_ = 3.4; *p* = 0.023; η^2^ = 0.15], and hip flexion [*F*_(2.0,54.1)_ = 21.9; *p* < 0.01; η^2^ = 0.55]. However, no statistically significant effect of approach distance was observed upon joint angles and tibia angle [*F*_(1,22)_ ≤ 3; *p* > 0.1; η^2^ ≥ 0.13]. *Post-hoc* comparison revealed that the ankle joint was less dorsiflexed in the 20-m 45° plant step compared with all other CODs performed from the same distance. The knee flexion was decreased in the 45° CODs from both 4 and 20-m compared to all the CODs. Hip abduction in the 4-m 45° was decreased compared to the 4-m 90 and 180° CODs. Hip flexion was found to be increased in the 4-m and 20-m 180° CODs compared to the other CODs from the same distance. Hip flexion in the 20-m 135° COD was increased compared with the 45 and 90° CODs from the same distance (Figure 4). Tibia angle increased with each increasing angle of COD (Figure 4). No statistically significant interaction effects (angle × distance) were observed for any of the joint angles measured [*F*_(1,22)_ ≤ 2.58; *p* > 0.09; η^2^ ≥ 0.19].

### Force vs. Velocity Dominant COD Muscle Activity

**Figure 5:**
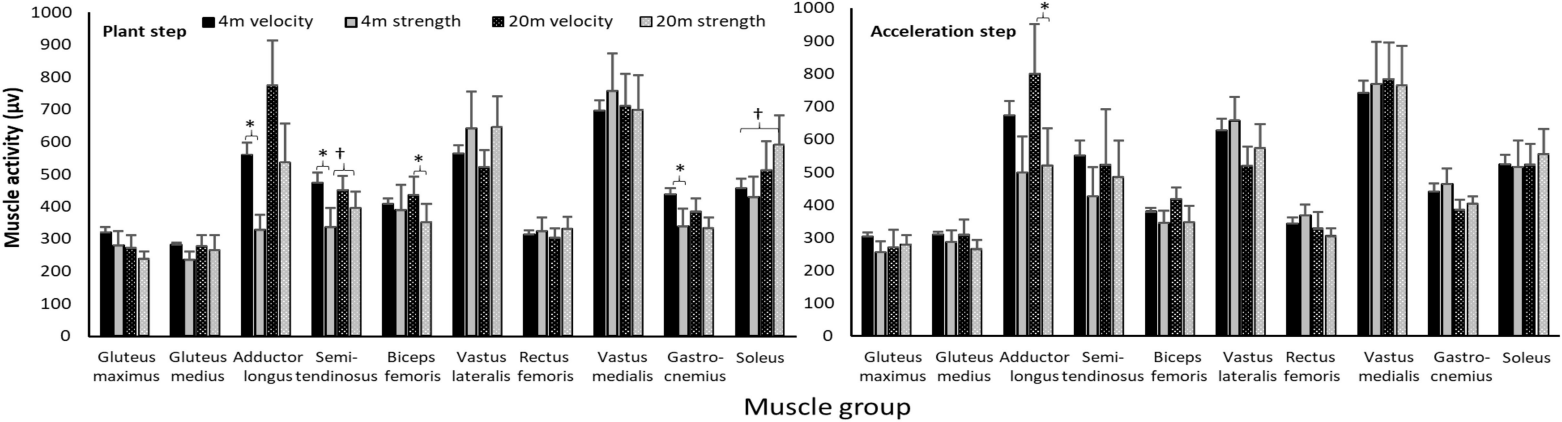
Comparison of peak (±SEM) muscle activity in the COD and acceleration step when performing velocity- and force-dominant CODs with 4 and 20-m sprint approaches. ^*^indicates a significant difference in muscle activity between these two force- and velocity-dominant CODs with this approach on a *p* < 0.05 level. ^†^indicates a significant difference in muscle activity between these two approaches on a *p* < 0.05 level.

When the angles of the CODs were categorized as force and velocity CODs, a significant effect was found for the adductor longus, semitendinosus, gastrocnemius, and biceps femoris between force and velocity CODs [*F*_(1,22)_ ≥ 6.5; *p* ≤ 0.034; η^2^ ≥ 0.45]; in the plant step and in the acceleration step, a significant effect was found only for the adductor longus [*F*_(1,22)_ = 7.1; *p* = 0.028; η^2^ = 0.47]. In addition, a significantly higher muscle activation was found only when the approach was with 20-m compared with 4-m for the soleus muscles [*F*_(1,22)_ = 7.1; *p* = 0.029; η^2^ = 0.47] and a significant interaction effect for the semitendinosus [*F*_(1,22)_ = 5.5; *p* = 0.047; η^2^ = 0.41] in the plant step (Figure 5). *Post-hoc* comparison revealed that higher muscle activation was observed when performing velocity CODs compared with the force CODs for all four muscles, especially with the 4-m approach (Figure 5, Table 2).

**Table 2:** Peak (±SD) EMG activity for the different muscles during the plant and acceleration step at the different angles and with 4- and 20 m approach.

	**Gluteus maximus**	**Gluteus medius**	**Adductor longus**	**Semitendinosis**	**Biceps femoris**	**Vastus lateralis**	**Rectus femoris**	**Vastus medialis**	**Gastrocnemius**	**Soleus**
**PLANT STEP**										
**4 m approach**										
45°	340 ± 211	264 ± 105	816 ± 564	391 ± 211	394 ± 229	546 ± 269	312 ± 144	693 ± 364	508 ± 255	441 ± 269
90°	257 ± 100	274 ± 90	465 ± 368	403 ± 190	421 ± 224	676 ± 314	312 ± 173	751 ± 364	364 ± 214	483 ± 397
135°	245 ± 164	208 ± 84	352 ± 204	368 ± 244	360 ± 258	623 ± 375	320 ± 110	730 ± 371	293 ± 230	477 ± 276
180°	283 ± 149	247 ± 116	306 ± 152	314 ± 193	349 ± 236	627 ± 357	298 ± 160	787 ± 418	383 ± 170	386 ± 160
**20 m approach**										
45°	276 ± 127	295 ± 118	897 ± 452	499 ± 183	471 ± 286	498 ± 222	345 ± 150	744 ± 375	392 ± 139	553 ± 300
90°	242 ± 151	268 ± 135	762 ± 520	444 ± 162	396 ± 185	574 ± 239	268 ± 91	686 ± 307	374 ± 166	497 ± 334
135°	221 ± 76	274 ± 240	678 ± 526	459 ± 256	352 ± 127	660 ± 420	275 ± 95	672 ± 415	385 ± 236	504 ± 273
180°	244 ± 84	273 ± 119	474 ± 354	373 ± 161	313 ± 201	593 ± 315	381 ± 199	753 ± 339	293 ± 119	673 ± 419
**ACCELERATION STEP**										
**4 m approach**										
45°	285 ± 110	313 ± 104	737 ± 490	589 ± 634	378 ± 130	613 ± 380	292 ± 171	767 ± 442	463 ± 310	533 ± 282
90°	309 ± 205	284 ± 82	687 ± 585	512 ± 485	366 ± 180	761 ± 416	355 ± 172	807 ± 392	434 ± 248	538 ± 383
135°	266 ± 114	248 ± 70	636 ± 540	453 ± 493	336 ± 155	701 ± 381	331 ± 123	781 ± 429	464 ± 223	555 ± 320
180°	253 ± 129	291 ± 132	411 ± 267	397 ± 173	351 ± 118	617 ± 229	383 ± 111	779 ± 439	464 ± 148	490 ± 245
**20 m approach**										
45°	216 ± 68	286 ± 156	933 ± 553	804 ± 770	422 ± 122	517 ± 243	358 ± 235	808 ± 446	395 ± 86	508 ± 163
90°	288 ± 208	336 ± 207	750 ± 562	531 ± 431	392 ± 150	557 ± 203	286 ± 152	785 ± 348	368 ± 134	543 ± 295
135°	294 ± 129	260 ± 106	611 ± 398	541 ± 419	365 ± 157	627 ± 318	291 ± 103	753 ± 408	411 ± 67	487 ± 209
180°	273 ± 107	262 ± 94	514 ± 446	525 ± 511	346 ± 185	548 ± 324	309 ± 103	800 ± 392	408 ± 152	619 ± 385

## Discussion and Implications

The aim of this study was to compare different CODs from different approach distances with each other to investigate how the suggested strength- and velocity demands affects step and joint kinematics and muscle activation. The main finding was that increased angle of the COD maneuver increased COD time, change in vertical center of mass displacement during the plant step, contact times, number of deceleration steps, and flexion/abduction of the joints, and tibia angle during the plant step. The 20-m approach distance affected only center of mass displacement and number of deceleration steps, which increased. When CODs were categorized as force- and velocity-dominant, a higher muscle activation for the adductor longus, semitendinosus, gastrocnemius, and biceps femoris muscles was observed when performing velocity-dominant CODs compared with the force-dominant CODs for all four muscles, especially with the 4-m approach.

COD is a multiple-step maneuver (Andrews et al., 1977), where deceleration is an important consideration for overall COD performance (Spiteri et al., 2013; Dos'Santos et al., 2018). Deceleration requirements were hypothesized to be larger when approaching the COD from 20-m. This was confirmed as the 20-m COD required a greater sum of deceleration steps prior to the plant step compared to the 4-m counterpart, distributing deceleration forces over more steps (Jones et al., 2016). Furthermore, 20-m CODs revealed a greater displacement of the center of mass, which probably was the result of a higher center of mass while running toward the COD. The joint angles, tibia angle, and contact time in the plant step were equal when comparing 4-m vs 20-m CODs (Figure 3), indicating that technique and force produced in the plant step were unaffected by approach distance. This is logical, because a subject has a certain strength to maintain body position when changing direction, whereby a reduction in velocity is necessary to manage the COD task and avoid excessive knee joint loading (Besier et al., 2001). Furthermore, friction with the floor sets the limits of how much the subject can lean with a certain strength for optimal force production by avoiding rotation and stumbling due to momentum (Luo and Stefanyshyn, 2011). By leaning and simultaneously increasing the distance of center of pressure from the lateral foot plant, relative to the center of mass, the forces produced will be more horizontal, which is more advantageous in sharper angle COD (Havens and Sigward, 2015). Therefore, a far too upright position, producing more vertical ground reaction force, might limit net forces in the appropriate direction when turning with sharper angles (Dayakidis and Boudolos, 2006). As such, participants must have the same joint angles in the plant step independent of the sprint approach, making the velocity adjustments in the deceleration phase crucial, by decreasing the velocity to a force the subject can tolerate. Greater eccentric strength has been associated with a faster COD performance as stronger athletes are more able to decelerate from faster approaching velocities (Jones et al., 2017). Jones et al. (2017) also introduced the concept of a “self-regulation” effect of approaching velocity by adjusting the velocity prior the plant step to a load which the athlete can handle. The approaching velocity is dictated by the angle of the turn, where sharper cuts (>60°) requires substantial braking, while velocity maintenance is key throughout the COD for the <45° turns (Hader et al., 2015; Jones et al., 2019). In between, (45–60°) requires moderate braking (Dos'Santos et al., 2018).

Muscle activation was not significantly different in the plant step when comparing 4 and 20-m CODs, except for the semitendinosus and soleus, which were higher in the force-dominant CODs from 20-m. A greater hip flexion in the force-dominant CODs from 20-m, although not significant, might account for the higher muscle activity in the semitendinosus (Figure 4). A greater hip flexion in the plant step requires more concentric force of the semitendinosus to extend the hip in the propulsive movement of the plant step, although there is a lack of data to confirm this. With increased angles of the COD turn, the contact time, joint angles, and displacement of the center of mass were found to increase. The velocity-dominant CODs showed less hip and knee flexion (Figure 3) and greater knee joint flexion angles increases contact times (Dai et al., 2015). Also, resisting hip flexion has been found to be associated with a faster COD performance (Welch et al., 2019). The velocity-dominant CODs was furthermore accompanied with more vertical force production due to a higher center of mass (Schreurs et al., 2017). The 180° COD in this study was found to be >0.25 s, the only COD maneuver where the plant step was not reliant on a fast stretch-shortening cycle (Aagaard et al., 2002; Flanagan and Comyns, 2008).

The only difference in muscle activity when comparing the CODs at different angles was observed in the adductor longus muscle. When performing cutting movements, athletes tend to rotate their trunk toward the direction of travel prior to the plant step (Mornieux et al., 2014), where the adductors function as hip stabilizers (Neptune et al., 1999; Bencke et al., 2013). Given the peak muscle activity observed for the adductor longus, it seems to be more important for stabilizing the hip in velocity-dominant CODs, which might be explained by higher velocities inducing shorter range of motion to provide force within a short time frame. This explanation is accompanied by the joint kinematic data where less hip abduction was observed in the velocity-dominant CODs. However, when divided into force- and velocity-dominant CODs, muscle activity was found to be higher in the velocity-dominant CODs for the adductor longus, semitendinosus, biceps femoris, and gastrocnemius (Figure 4).

A possible explanation for this can be that the velocity-dominant CODs were performed at higher velocities, that may cause increased eccentric loading. Higher running velocities may cause greater potentiation due to a faster pre-stretch in a fast countermovement, building up the muscles' level of active state by attaining more cross-bridges before contraction (Bobbert et al., 1996). Dietz et al. (1979) suggested that a pre-stretch at a higher muscle-contraction velocity also triggers spinal reflexes, which induces higher muscle stimulation. With shorter contact times at greater running speeds, the functional role of the reflexes might become even more important for powerful force productions, as pre-stretching the muscle absorbs energy that is temporarily stored in series of elastic elements before being re-utilized in the concentric contraction (Bobbert et al., 1996). As such, the time to produce the necessary force to change momentum in the velocity-dominant CODs is shorter than in the force-dominant CODs, as observed by the shorter contact times. Kyröläinen et al. (1999) pointed out that shorter contact times at higher velocities demand a higher level of pre-activation for timing muscular actions, which increases muscle activity in the eccentric braking phase of the stance. While being eccentrically lengthened, the gastrocnemius induces high muscle activation and resistive force, where motor units are restricted to a short period of time (Nardone et al., 1989), with pre-activity increasing linearly with increasing running speed (Komi et al., 1987). A study by Hobara et al. (2007) on repeated hopping suggested muscle activity of the gastrocnemius is related to leg stiffness, which increases when the ankles resist being dorsiflexed to produce shorter contact times. This might also be the case for the current study, as the gastrocnemius is important for absorbing the impact in a COD by plantar flexing the ankle (Neptune et al., 1999). At higher velocities and impact loads, more muscle activity is required of the hip and knee extensors as well (Neptune et al., 1999).

The hamstring muscles play a key role in decelerating knee-joint moments to prevent inappropriate knee-joint loading during foot contact when performing a COD (Mclean et al., 2004, 2005; Bencke et al., 2013). Such moments seem to be greater in the velocity-dominant CODs due to a higher movement velocity to change the direction, as shown by the shorter test times (Rau et al., 1997). This finding of increased hamstring activity did not to follow the findings of Hader et al. (2016), who found that with increasing angle of COD hamstring activity increased. However, they only investigated hamstring activity between 45 and 90° and not with force-dominant CODs and thereby make it difficult to compare it with the present study.

This study has some limitations. Firstly, no force plate was included in the plant step, which could offer more insight about the forces that occur when performing the different CODs. Secondly, there is always an inherent risk of cross-talk between neighboring muscles using EMG measurements, especially in fast movements like CODs (Winter et al., 1994). Future studies should include a force plate to investigate the forces involved in the different CODs, in addition to EMG measurements of the penultimate step. Furthermore, although familiarization was required a priori testing, future studies should utilize multiple attempts to increase reliability. In this study the highest EMG activity of the muscles recorded were sampled from the plant step and acceleration step. Since we did not include exact timing of these maximal EMG activity during these two steps it is not possible to state if the muscle activity stems from eccentric or concentric muscle actions and therefore must be interpreted with caution.

## Conclusion

Approach distance affects the deceleration required for velocity adjustments to complete the COD maneuver, while the technique in the COD and acceleration steps remain unaffected. The velocity-dominant CODs revealed higher muscle activity in the plant step for the adductor longus, semitendinosus, biceps femoris, and gastrocnemius, with less flexion/abduction in the hip, knee, and ankle joint. The higher observed muscle activity was probably due to a higher eccentric loading when performing velocity-dominant CODs, as shown by the shorter contact times. Based upon the findings of the current study we suggest as practical application that for enhancing of COD performance at smaller angles (<90°) training exercises should be performed at a high velocity that require high peak muscle activation and fast stretch-shortening cycle (SSC), this can be accomplished utilizing plyometric exercises such as hurdle jumps and drop jumps. To develop CODs with larger angles (90°) movement-specific exercises that require greater the hip- and knee joint flexion exercises are recommended such as squat variations utilizing a slow SSC. Furthermore, eccentric training to develop braking capabilities might be more applicable for developing CODs of greater angles (>45°) and approaching distances due to greater braking requirements. Future studies that include these exercises should be performed to investigate if these exercises, based on the present findings, target these different CODs specifically or not.

## Data Availability Statement

The raw data supporting the conclusions of this article will be made available by the authors, without undue reservation.

## Ethics Statement

The studies involving human participants were reviewed and approved by Norwegian Center for Research Data. The patients/participants provided their written informed consent to participate in this study.

## Author Contributions

HF and HR conducted the data collection and analysis and wrote the first draft, while RT helped with the data analysis, revisions to the manuscript, and supervision of the project. All authors contributed to the article and approved the submitted version.

## Conflict of Interest

The authors declare that the research was conducted in the absence of any commercial or financial relationships that could be construed as a potential conflict of interest.
